# Capturing the pulse: a state-of-the-art review on camera-based jugular vein assessment

**DOI:** 10.1364/BOE.507418

**Published:** 2023-11-28

**Authors:** Coen Arrow, Max Ward, Jason Eshraghian, Girish Dwivedi

**Affiliations:** 1 School of Medicine, University of Western Australia, Perth, Australia; 2 Advanced Clinical and Translational Cardiovascular Imaging, Harry Perkins Institute of Medical Research, University of Western Australia, Perth, Australia; 3 Department of Computer Science and Software Engineering, University of Western Australia, Perth, Australia; 4 Department of Electrical and Computer Engineering, University of California (Santa Cruz), California, USA; 5 Department of Cardiology, Fiona Stanley Hospital, Perth, Australia; 6 coen.arrow@research.uwa.edu.au; 7 girish.dwivedi@perkins.uwa.edu.au

## Abstract

Heart failure is associated with a rehospitalisation rate of up to 50% within six months. Elevated central venous pressure may serve as an early warning sign. While invasive procedures are used to measure central venous pressure for guiding treatment in hospital, this becomes impractical upon discharge. A non-invasive estimation technique exists, where the clinician visually inspects the pulsation of the jugular veins in the neck, but it is less reliable due to human limitations. Video and signal processing technologies may offer a high-fidelity alternative. This state-of-the-art review analyses existing literature on camera-based methods for jugular vein assessment. We summarize key design considerations and suggest avenues for future research. Our review highlights the neck as a rich imaging target beyond the jugular veins, capturing comprehensive cardiac signals, and outlines factors affecting signal quality and measurement accuracy. Addressing an often quoted limitation in the field, we also propose minimum reporting standards for future studies.

## 1. Introduction

Heart failure (HF) is a significant health problem worldwide. In high income countries, HF is the most common diagnosis for hospital admissions for those over 65 [1]. The 10-year survival probability for those with the diagnosis is less than 30% [2], and the reported physical quality of life for those individuals is poor [3]. Beyond the individual effects, the economic burden for the condition is high, with over $ 108 billion spent globally, and the current upward trajectory of global disease burden is expected to continue with the aging population [4–6]. The dominant burden is due to hospitalisations, costing both time for the patient, and money for the health system [4]. As a chronic, progressive disease, the accumulated burdens on the body over time result in frequent rehospitalisation for those diagnosed with HF, with 25% likely to be re-admitted after 30 days, and 50% likely to be re-admitted within 6 months, with a large percentage of these being avoidable [7,8]. Elevated central venous pressure (CVP) has been associated with rehospitalised patients both when presenting at the hospital [9], as well as the increase in time-integrated pressure out of hospital [10], suggesting this may be used as a marker for early intervention, reducing rehospitalisations [11]. Development of an at-home, non-invasive measurement of this pressure may be beneficial in this domain, as some individuals have shown interest in the use of mobile health applications to manage their own symptoms [12].

Technological advancements in the remote monitoring of patients includes wearable technologies such as flexible sensors that can be used to detect cardiorespiratory signal [13,14]; implanted devices such as the continuous glucose monitors [15]; and the use of cameras and computers in telehealth consults. The use of cameras in physiological monitoring is a popular area of research, and companies are emerging using artificial intelligence (AI) and smartphone technologies to provide consumers with information to help guide self-management of their conditions [16,17]. One such measure, concerning HF, is the potential use of cameras in the replication of a common bedside technique in the inspection of the jugular venous pulsations (JVP). The jugular veins act as a manometer to the right heart, and an increase in the height of a visible pulsation on the neck can indicate an increase in CVP [18]. CVP measurements are pivotal in assessing volume status and right heart function in HF patients, guiding diuretic and vasodilator therapy. Persistently elevated CVP may indicate the need for advanced interventions, such as ventricular assist devices or heart transplantation. Regular monitoring of this pressures aids in optimizing treatment efficacy, prognostication, and ensuring timely therapeutic adjustments. In hospitals, clinicians rely on CVP measurements (acquired either through the invasive gold standard method using a central catheter or via bedside examination) to tailor patient therapy. Bedside examinations are difficult to perform, even for highly trained clinicians, with the subtle pulsations often being difficult to visualise, especially in patients with higher body mass index (BMI) [19]. After discharge, utilizing the invasive method becomes impractical. Instead, patients primarily rely on self-reported weight measurements to determine fluid overload levels. To enhance remote monitoring capabilities and promote patient independence, recent innovations have turned to video assessments. Two studies have demonstrated that the accuracy of clinicians’ CVP estimations via recorded videos are comparable with traditional bedside examinations [20,21]. Despite this, the role of video assessment for the purpose of CVP estimation is not yet standard practice in either remote or clinical settings.

The field of physiological assessment by video is broad, and in the process of establishment. From recent large-scale surveys and systematic reviews [22–29], a consistently mentioned challenge was the lack of standardization in studies and reports. To address this, this review outlines factors likely to affect the accuracy of estimations, from both system and study design, suggesting reporting guidelines for future investigations. Furthermore, much of the literature is centred around the face as a region of interest (ROI), for the purposes of measuring heart rate. This review highlights the benefits of imaging the neck, which provides a richer source of information as it contains information relating to both the right and left sides of the heart through the carotid arteries and jugular veins. Additionally, the neck, as compared with the face, contains fewer personally identifiable features, reducing the difficulties in the compilation of large publicly available datasets.

In this review, we begin with a brief introduction to the mathematical model for cameras used to motivate the variety of different approaches taken. We then provide systematic review of the approaches taken in the literature that use video and signal processing in the assessment of the jugular veins and the associated results. The discussion consists of a meta-review, identifying the diversity of approaches in camera-based physiology assessments, and the general trend of these effects, providing some guidance in hardware selection and considers the study design for experiments such as this, and factors that need to be considered. We then summarise the literature in terms of general design implementation and provide future direction regarding reporting of these results for future reviews and meta-analyses.

## 2. Modelling the camera signal

The typical model for the detected signal is based on the skin reflection model and is found in numerous publications [26,30,31]. Variations in surface angles and underlying tissue over the physiological signals of interest give rise to different methods of signal acquisition. Regions where motion is expected, such as pulsatile motion in the neck or wrist due to the pumping of the heart or the rise and fall of the chest and shoulders due to breathing, may lead some investigators to design imaging systems that emphasise either the specular or diffuse components of reflection, depending on the signal of interest. With this in mind:

The signal as measured by each pixel at time t can be expressed as:

(1) $$I_{\text{pixel}}\left(t\right)=\int_{-\infty }^{\infty }Q_{\text{efficiency}}\left(\lambda \right)I_{\text{sensor}}\left(t,\lambda \right)d\lambda +N_{\text{sensor}}\left(t\right)$$

Where $I_{p}$ is the pixel value, $Q_{\text{efficiency}}\left(\lambda \right)$ is the quantum efficiency of the pixel with wavelength λ , $I_{sensor}$ is the irradiance (power received by the pixel sensor) and $N_{\text{sensor}}$ is the noise of the sensor. If the signal is being received from a surface at distance d from the pixel, assuming no losses of intensity due to the air, the relationship between the irradiance of the surface, and that detected by the pixel is:

(2) $$I_{\text{sensor}}(t,\lambda )=\frac{A}{4{\pi d}^{2}}\int_{\theta_{1}}^{\theta_{1}}I_{\text{surface}}(t,\lambda ,\theta )d\theta$$

Where A is the size of the sensor, and $I_{\text{surface}}$ is the emitted irradiance of the surface that corresponds with the light received over the region A , as determined by the angle θ . Based on Shafer’s dichromatic reflectance model [32], we can decompose the emitted light from the surface in terms of the types of reflected light, either specular $r_{\text{specular}}$ or diffuse $r_{\text{diffuse}}$ .

(3) $$I_{\text{surface}}(t,\lambda ,\theta )=I_{\text{incident}}(t,\lambda ,\theta )(r_{\text{specular}}(t,\lambda ,\theta )+r_{\text{diffuse}}(t,\lambda ,\theta ))$$

Specular reflection is like that of a mirror, where the angle of incidence is equal to the angle of the reflected light, when measured from the normal of the surface. As such, a rough surface will reflect incident light in a variety of directions compared with the macro-surface orientation. On the aggregate, this can be considered diffuse reflection, but in this instance, we will consider this the diffuse component of specular reflection. The overall amount of light reflected in a specular fashion is relatively low, between 2 - 4% [33]. The diffuse component of this is even smaller, between 2.67 - 9.91% of total reflected light depending on factors such as skin roughness and the angle of incidence of the incoming light [34]. Although the total proportion of specular reflection is small, relative to that of diffuse reflection, the solid angle over which it is spread is small, and so may contribute a larger part of the signal as measured by the camera.

The diffuse reflective component at a point, $r_{\text{diffuse}}(t,\lambda ,\theta )$ , is due to the initial transmission of light beneath the surface. After transmission below the tissue surface, the light interacts with matter either by absorption or scattering. Some proportion of light is then scattered back out from the surface. The angle of propagation from the normal of the surface is random, and so is more accurately described as diffuse. The likelihood of a photon transmitting through the skin surface and being reflected back is dependent on the incident wavelength of the photon. Longer wavelengths generally correspond with increased likelihood of transmission.

For jugular venous pulsations, the measured signal has a large time-varying component due to changes in both specular and diffuse reflections from the underlying blood vessels. The pulsating vein gives rise to a time dependence of the angle of the surface, and the varying amount of venous blood with the cardiac cycle results in a time varying diffuse reflection component. Design considerations are expanded on in the discussion section and include the use of specific light wavelengths known to be less penetrating to maximise specular reflection, or polarizing filters to maximise diffuse reflection.

## 3. Methods

**Fig. 1.**
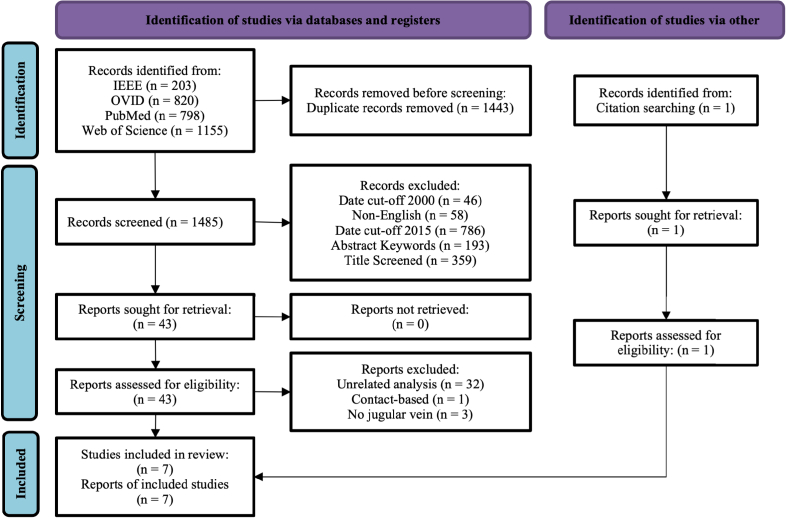
PRISMA Systematic Review Flowchart

This systematic review adheres to the PRISMA (Preferred Reporting Items for Systematic Reviews and Meta-Analyses) guidelines [35]. The PRISMA flowchart, which outlines the progression of studies through the review, can be found in Fig. 1. Below, we detail the study selection process to facilitate the reproduction of results in future reviews.

### 3.1 Eligibility criteria

As is typical in PRISMA systematic reviews, we have used the PICO Framework to structure the eligibility of studies included in the review [36]. All Participants were included. The Interventions were restricted to the use of cameras for the assessment of the jugular veins. The review did not limit studies based on Comparison devices or Outcome measures. Studies were from database inception through to October 2022, limited to English, including all conference papers and journal articles. All included manuscripts are published and peer reviewed. As most published literature for this topic consists of proof-of-concept works, and the literature is relatively sparse (n = 7), all studies were included.

### 3.2 Information sources

Data was acquired through searches of the OVID, Web of Science, IEEE and Pubmed databases, from inception up to October 2022. References from prior systematic reviews of camera based measurement of physiology and vital signs by McDuff *et al.* [23], Pham *et al.* [24], Selvaraju *et al.* [25] and Zaunseder *et al.* [26] were included.

### 3.3 Search strategy

All databases were searched with ("Camera" OR "iPPG" OR "PPGi" OR "Photoplethysmography") AND ("Carotid" OR "Jugular" OR "CVP" OR "JVP" OR "JVD" OR "Neck") with the search augmented for each database accordingly.

Searches included the carotid artery as any assessment of the vasculature in the neck may have included jugular venous assessments.

### 3.4 Selection process

The collected items were collated and added to Endnote for de-duplication. From the identification of the first investigation in the use of cameras for Photoplethysmography (PPG), also known as remote Photoplethysmography (rPPG) by Wu, Blazek and Schmitt [37], in 2000, a first date cut-off was used. Non-English items were then removed. Titles and abstracts were screened chronologically, to determine the first published author using cameras for the neck pulsation assessment [38], and a date cut-off for all other articles was then applied, removing all items prior to 2015. Articles were then removed if they did not contain the word "camera" in the abstract, and then again if they did not contain "jugular", "carotid" or "neck". Titles were then screened for eligibility. Abstracts were then manually screened, and accepted articles retrieved for synthesis.

### 3.5 Data items and collection process

For each article, the primary interest of the review is around both study and system design. Study design includes participant demographics, setting and setup information in the data acquisition process, and the reference standard used. System design was separated into hardware, and software, with software further divided into image and signal processing methods.

## 4. Experimental setup and aims

**Fig. 2.**
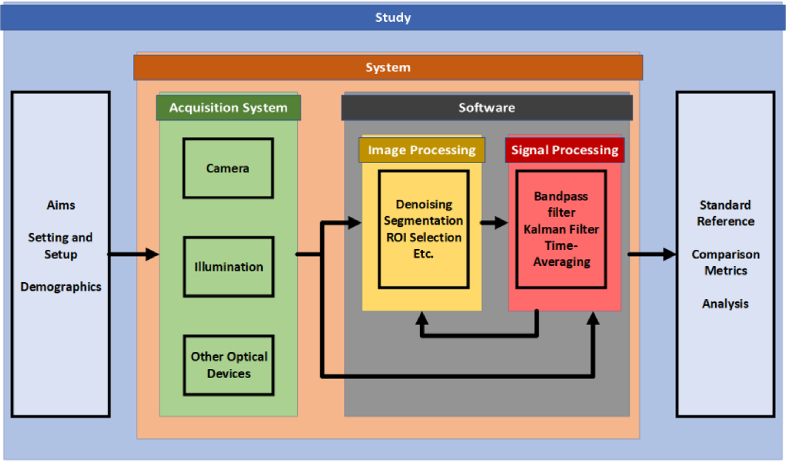
Overview of Study Design. The design of a study consists of both the system itself, and the environment where it is applied.

**Fig. 3.**
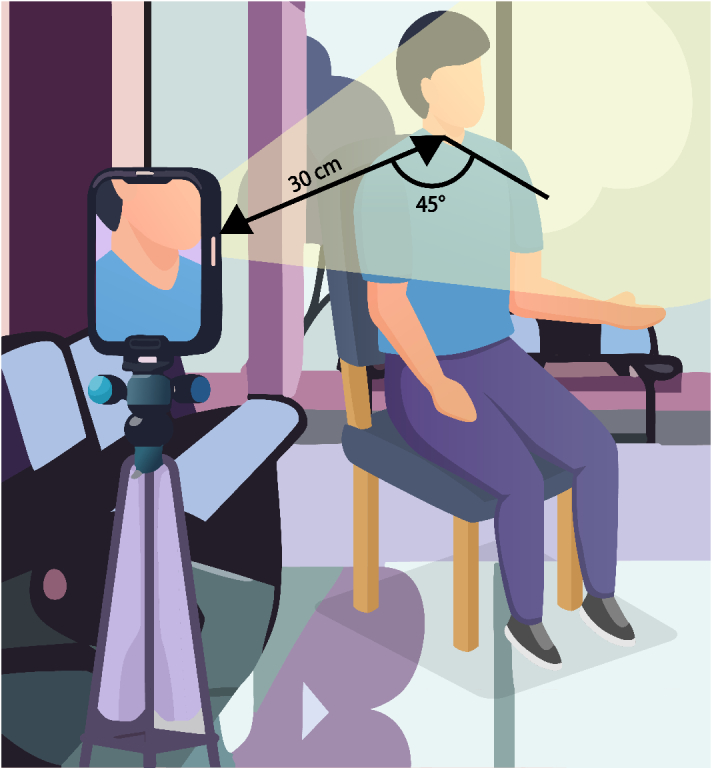
An illustration of the setup described Table 5. The setting of the study was in the emergency department of the hospital in Argentina. The participant is in a seated position, facing forward. The camera on the phone is situated at a distance of 30 cm from the neck, at an angle of 45°from the front plane of the person. The flash of the light from the phone is used to illuminate the neck. Adapted from *vecteezy.com*

Each study has been analysed in two parts – study design and system design (Fig. 2). Study design includes the demographic, location, setup, and standard reference information used and overall accuracy of the method. System design is primarily concerned with the hardware and software components used as a means of assessing the state-of-the-art system. We fabricate an illustrative in Table 5 with a figure showing a potential setup in Fig. 3. The path from the acquisition of the data through the software is not always consistent and may bypass signal processing methods altogether. We first give an overall description of the relevant demographic and setup of the studies. As the sample size of studies included in the review is relatively small (n = 7), with each employing unique setups, hardware and processing algorithms, we do not dwell on specific experimental design choices made by the investigators. In sections 6.3 and 6.5, we discuss in detail the potential pros and cons of specific design decisions to assist investigators in future studies.

### 4.1 Study design

#### 4.1.1 Study aims

The jugular veins provide two significant sources of clinically relevant information. The highest point on the neck of visible pulsation, relative to the sternal angle, correlates well with the CVP, and the waveform is associated with a range of cardiovascular diseases [39]. As such, investigators have tended to concentrate their efforts on augmenting videos as an assistive device for the clinicians [20,38], or visualising the waveform itself [31,40–42]. Moço *et al.* investigated the importance of ROI location and patient positioning [41]. Amelard *et al.* investigated the relationship between attenuation of infrared light in the jugular venous region, rather than concentrating on pulse wave extraction, although this was still included in the video processing system [43].

#### 4.1.2 Demographics

A summary of demographic data is shown in Table 1. All investigations consist of small pilot studies, ranging from 4 to 48 participants. The age range is from 9 – 75+ years, though only one study had a reported mean participant age over 30 [20]. In the context of HF, where JVP assessment is most common, patients are frequently in the older demographic. As such, future investigations may do well to include older participants to ensure accuracy on the cohort that is most likely to benefit from the technology. No study reported the skin tone or ethnicity of the participants, though one study described it as "varied". It’s important to highlight that skin tone is of particular importance when it comes to ensuring an equitable system. This is discussed further in section 6.3. Amelard *et al.* noted that the signal recorded for participants who are older, and with higher BMIs have a lower signal-to-noise ratio for the measured waveform [31]. This suggests that BMI (or other body morphology metrics) are of importance, and should be noted during experiments.

**Table 1.** Summary of Demographic Information for the included studies.

Investigator	Demographic Information					
	n (M/F)	Age Range ( μ±σ )	Skin Tone	Body Morphology	Pathology Status or Related Information	Hydration Status
Dang *et al.* 2015 [38]	5 (5/0)	22 – 48 (-)	-	-	-	-

Amelard *et al.* 2017 [31]	24 (13/11)	9 - 60 (28.7 ± 12.4)	-	Body Fat (%): 10.5 - 42.3 (21.0 ± 7.9)	-	-
				Muscle (%): 31.0 - 53.9 (40.4 ± 5.3)		
				BMI ( $kg.m^{-2}$ ): 16.4 - 35.1 (25.5 ± 5.2)		

Lam-Po-Tang *et al.* 2018 [40]	19 (12/7)	21 - 63 (25.5 ± 9.4)	-	BMI ( $kg.m^{-2}$ ): 16.2 - 25.6 (22.5 ± 2.3)	Healthy	-

Moço *et al.* 2018 [41]	4 (3/1)	28 – 41 (-)	-	Non-obese	-	-

Abnousi *et al.* 2019 [20]	48 (Mostly Male)	67 – 73^*a*^ (69.9 ± 8.04)	Variety (unspecified)	BMI ( $kg.m^{-2}$ ): 26.3 – 32.3* (29.6 ± 4.87)	Smoking Status: Never/Former/Current 37/7/4	-
				Chest Circumference (cm):		
				112.5 – 129^*a*^ (119.6 ± 12.01)		

Amelard *et al.* 2021 [43]	20 (10/10)	- (24 ± 4)	-	Height (cm): - (169 ± 11)	Healthy	2 L water consumed
				Mass (kg): - (66 ± 13)		

Saiko *et al.* 2022 [42]	10 (-)	-	-	-	Healthy	-

BMI: body mass index

^*a*^ Interquartile range reported

#### 4.1.3 Setting and setup

Setting and setup information is summarised in Table 2. Herein, we describe the range of experimental design decisions. The potential implications of these particular design considerations is elaborated on in sections 6.3 and 6.5.

**Table 2.** Summary of Experimental Setup Information for the included studies.^*a*^

Investigator	Setup Information					
	Setting	Patient Position	Camera Position Relative to Participant	Illumination Position Relative to Participant	Other Setup Information	Primary Reference Standard(s)
Dang *et al.* 2015 [38]	Lab	Supine, angled at the clinician’s direction	Distance: -Angle: -	-	-	Clinician
Amelard *et al.* 2017 [31]	Lab	Supine (0°)	Distance: 1.5 mAngle: Orthogonal to skin	Distance: 1.5 mAngle: Orthogonal to skin	-	Finger PPG Ultrasound
Lam-Po-Tang *et al.* 2018 [40]	Lab	Supine (45°)	Distance: 30 cmAngle: 45°	Distance: 30 cmAngle: 45°	-	Laser distance meter ECG
Moço *et al.* 2018 [41]	Lab	Supine (0°)Recumbent (45°)Sitting (90°)	Distance: -Angle: Orthogonal to skin	Distance: -Angle: -	-	Finger PPG Ultrasound
Abnousi *et al.* 2019 [20]	Hospital/Clinic	Recumbent (45°)Sitting (90°)	Distance: -Angle: Typical for bedside assessment	-	-	ClinicianRight Heart Catheter
Amelard *et al.* 2021 [43]	Hospital/Clinic	Supine (0°)Supine (-3°)Supine (-6°)	Distance: 1.5mAngle: Orthogonal to skin	Distance: 1.5mAngle: Orthogonal to skin	Negative angles used to increase pressure.Negative lower body pressure induced to lower pressure.Valsalva manoeuvre performed to increase pressure.	Right Heart Catheter
Saiko *et al.* 2022 [42]	Lab	Sitting (90°)	-	-	Baby oil applied to neck to increase specular reflection	Finger PPG

^*a*^ PPG: Photoplethysmography ECG: Electrocardiograph

All studies were conducted in either laboratory or clinical settings. Cameras were positioned at a variety of distances, from 30 cm up to 1.5 m. Not all studies reported the distance of the camera to the neck. All investigations used artificial lighting, though the specific spectra were not always clearly defined.

Most studies investigated participants in the supine position, mimicking the approach taken by clinicians by the bedside. One study investigated the effect of participant position and ROI selection on the neck, and so a small number of participants were assessed in various positions [41]. Another study assessed the change in the attenuation of the signal with experimentally induced pressure changes. These include tilting the body below the horizontal, increasing venous blood-flow to the head and torso, forced exhalation via the Valsalva manoeuvre to increase intra-thoracic pressure, and the application of lower body negative pressure, mimicking hypovolemia [43].

## 5. Results

### 5.0.1 Comparison and results

Results from the literature can be characterised as either quantitative or qualitative. Quantitative results provide insight into the timing and signal strength of the signals measured by the imaging system. Qualitative results provide hints towards improvement in setup and future processing algorithms. The results have been summarised in Table 3.

**Table 3.** Summary of the quantitative and qualitative for the included studies.^*a*^

Investigator	Quantitative Results			Qualitative Results	
	Reference Standard(s)	Metric	Results	Reference Standard(s)	Results
Dang *et al.* 2015 [38]	-	-	-	Clinician	The system could realise pulsatile regions in all subjects, as verified by the clinician.
Amelard *et al.* 2017 [31]	Finger PPG	Pearson Correlation	Jugular vein region: -0.73 ± 0.17 Carotid artery region: 0.85 ± 0.80	Ultrasound	Highly correlated regions were associated with the carotid artery and the jugular vein as confirmed by ultrasound.
		Time lag between arterial and venous waveforms.	427 ± 99 ms	Typical CVP Waveform	All waveforms extracted from the imaging system exhibited the main characteristics of the central venous waveform (c, x, v, and y waves)
Lam-Po-Tang *et al.*2018 [40]	Laser distance meter	Pearson Correlation	0.93 ± 0.05	Typical CVP waveform	All waveforms extracted from the imaging system exhibited all characteristics of the central venous waveform (a, c, x, v, and y waves)
	ECG	Time between R in the QRS complex, and v and c points on the CVP waveform.	v-R [min, max] = [346.5, 547.2] ms R-c [min, max] = [139.6, 248.3] ms		
Moço *et al.* 2018 [41]	-	-	-	Ultrasound	Videos and ultrasound were acquired in a variety of body positions. Significant changes in morphology of the jugular vein and waveform were noted, emphasising the importance of patient position.
Abnousi *et al.* 2019 [20]	Clinician and Right-Heart Catheter	Mean Error (95% confidence interval)	Bedside: -2.90 (-4.33 to -1.40) cmH_2_O	N/A	N/A
			Video: -1.84 (-3.22 to -0.46) cmH_2_O		
			Augmented video: -0.8 (-2.18 to 0.61) cmH_2_O		
Amelard *et al.* 2021 [43]	Right-Heart Catheter	Pearson Correlation Coefficient	Head-down-tilt: 0.94 [0.84, 0.99]*	-	-
			Negative Pressure: 0.85 [0.76, 0.92]*		
			Valsalva: 0.94 [0.85, 0.99]*		
Saiko *et al.* 2022 [42]	Publicly available waveforms [31]	SNR Comparison	Difference between maximum and minimum waveforms was used to compare SNR and this method increased SNR by 6 times.	Publicly available waveforms [31].	Comparison of timing between correlated and negatively correlated regions was found to be relatively accurate.
				ECG	ECG was used to confirm the generated timing of waveforms.

^*a*^ PPG: Photoplethysmography ECG: Electrocardiograph CVP: Central Venous Pressure

#### Quantitative results

Amelard *et al.* [31] visualised the dual pulsating regions in the neck and showed two regions with high Pearson correlation with a synchronously acquired finger pulse. The arterial (0.85 ± 0.8), and venous (-0.73 ± 0.17) waveforms respectively. Lam Po Tang *et al.* [40] utilised a displacement measurement algorithm to track skin motion and yielded comparable Pearson correlation between estimated motion and a laser distance meter (0.93 ± 0.05). Furthermore, temporal spacing between electrocardiogram (ECG) and CVP waveform characteristics were first established. Saiko *et al.* [42] used a novel imaging technique based on increasing specular reflection to show a 6-fold increase in SNR for the waveform measurements as compared with Amelard *et al.*’s initial PPG based approach [31]. SNR was calculated by the mean difference between the peak and the trough of the waveforms generated.

Abnousi *et al.* [20] demonstrated the feasibility of using both raw and augmented videos to estimate the CVP of participants by neck examination. Augmenting the video by enhancing motion within a specific frequency band decreased mean measurement error from -2.9 cmH_2_O at the bedside to -0.8 cmH_2_O with augmented videos. Amelard *et al.* [43] demonstrated high mean Pearson correlation (> 0.85) with DC variations of the PPG signal under experimentally induced pressure changes.

#### Qualitative results

Dang *et al.* [38] showed that it is possible to identify pulsating regions in the neck, as confirmed by clinicians. Amelard *et al.* [31] revealed the waveform of the jugular veins by remote photoplethysmography imaging. They also showed that the arterial and jugular pulsations in the neck are highly correlated with the arterial finger pulse, and that this can be used to distinguish between pulsating regions on the neck, confirmed by ultrasound. Positive correlation is associated with the carotid artery, and negative correlation with the jugular vein. Lam-Po-Tang *et al.* [40] used a technique previously employed to measure carotid artery waveforms, and showed that the CVP waveform can be acquired from measurement of skin displacement. Moço *et al.* [41] demonstrated the importance of patient positioning and region of interest selection for neck based assessments such that the same location on the neck can be dominated by either the carotid artery pulse or the jugular venous pulse depending on if the participant is seated, recumbent or supine.

### 5.2 System design

The ideal system will vary depending on the intended use-case. An ideal hardware system is dependent on the the system setup and the processing algorithms. Similarly, the existence of a benchmarking dataset for the purpose of defining a best set of processing algorithms is also yet to be compiled. As such, we cannot comment on whether the studies included in this analysis are better or worse than each other. However, we can draw on the lessons learned from face-specific rPPG approaches on the expected benefits for each of the setups. More detail regarding the lessons drawn from facial rPPG approaches is outlined in section 6.5, but we briefly relay some of the potential decisions behind design choices.

#### Hardware

As shown in Fig. 2, the hardware used can be divided into the camera, artificial illumination sources, and any other optical devices present. The hardware systems used are summarised in Table 4.

**Table 4.** Summary of Hardware Information for the included studies.^*a*^

Investigator	Camera						Illumination			
	Model	FPS	Colour Channels or Filter	Bit Depth	Resolution	Polarization	Description	Brightness	Spectral Information	Polarizing Filter
Dang *et al.* 2015 [38]	Primesense (Model not specified)	30	RGB	-	-	-	-	200 - 500 Lux	-	-
Amelard *et al.* 2017 [31]	FLIR GS3-U3-41C6NIR-C	60	850-1000 nm Filter (NIR)	N/A	2048 x 2048	N/A	250W Lamp	N/A	Tungsten Halogen	N/A
Lam-Po-Tang *et al.* 2018 [40]	FLIR USB3 Flea3, FL3-U3-13Y3M-C	90	Monochromatic (no filter)	10	1280 x 1024	N/A	Blue LED	N/A	Blue light	N/A
Moço *et al.* 2018 [41]	IDS mEye	20	RGB (only R used)	8	500 x 500	N/A	Fluorescent Lamp	N/A	Philips EnergyLight HF3319	N/A
Abnousi *et al.* 2019 [20]	Basler aCA1920-155uc	155	RGB	8	1920 x 1280	N/A	LED Light source	N/A	N/A	N/A
Amelard *et al.* 2021 [43]	FLIR GS3-U3-41C6NIR-C	60	IR	N/A	2048 x 2048	Circular Polarizing Filter	NIR LED (LZ1-10R702, LEDEngin)	N/A	940 nm (FWHM 40nm)	Linear Polarizing Filter
Saiko *et al.* 2022 [42]	Basler acA 2000-165uc	250	RGB (only R used)	N/A	512 x 512	N/A	Bright, diffuse light	Bright (unspecified)	N/A	N/A

^*a*^ RGB: Red, green, blue FPS: Frames per second NIR: Near-infrared IR: Infrared LED: Light emitting diode FWHM: Full-width half-maximum

#### Camera

A range of cameras have been used, including red, green and blue (RGB), infrared (IR) and monochromatic cameras, although not all colour channels were always used. It’s likely that the IR camera used by Amelard *et al.* [31,43] will extract signals from deeper within the neck, and so may increase the relative signal strength when compared with other colour channels. However, it’s noted that RGB cameras used by other investigators all managed to yield pulsatile signals. The frame rates for the videos were between 20 and 250 FPS. From Whittaker-Nyquist-Shannon sampling theorem [44–46], a frame rate of a minimum of 8 FPS is required to accurately reconstruct a cardiac cycle. Higher frame rates will likely yield better results, and so higher frame rates are preferred where possible. Resolutions ranged from 500 x 500 to 2048 x 2048. Given a fixed distance from the participant, an increase in resolution will result in a larger number of pixels within the ROI, likely increasing the overall SNR. A near infrared (NIR) wavelength bandpass filter was used by Amelard *et al.* [31].

#### Illumination

All studies used artificial illumination sources. This included light emitting diode (LED) arrays in the blue and NIR ranges, fluorescent and halogen lamps, and otherwise unspecified lighting. Lighting is of particular importance when considering the skin reflection model illustrated in section 2. Increasing the incidence of photons on the neck will yield a larger measured signal by the camera. Blue LEDs are used to emphasise the surface of the skin (specular reflection), and longer wavelengths may emphasise the pulsatile blood via diffuse reflection. The best lighting for each situation will depend on the intended method of pulse extraction, as is evident by the contrast in approaches taken by Saiko *et al.* [42], and Amelard *et al.*[31,43], who primarily focus on the extraction of specular and diffuse reflections respectively.

#### Other hardware

Polarizing filters were used by Amelard *et al.*[43] to enhance PPG signals at depth. Diffusers were used for illumination sources in some instances [31,42].

### 5.3 Software

Image processing techniques consist primarily with spatial processing algorithms for selection and refinement of ROIs. Spatial processing algorithms involve denoising and image segmentation. Signal processing is typically concerned with temporal signal processing, including time averaging, denoising and the use of frequency filters. Signal processing techniques can be used to refine ROIs beyond spatial techniques.

#### Image processing

*Pre-processing:* The averaging of a region of pixels (spatial averaging) reduces the effect of sensor noise. Spatial averaging is performed in nearly all approaches, typically with a Gaussian filter, but mean filters are also used [31,38,41–43]. One approach uses large overlapping regions, with the centre of the region being used as a "control point", for which signal processing if later performed [40]. Dang used a motion tracking algorithm to reject videos if undesired motion was detected above a threshold [38]. A novel calibration tool was used in conjunction with an IR camera to emphasise diffuse reflectance and the PPG signal, with specular reflective components minimised [43].

*ROI Selection and Refinement:* Manual segmentation of a region of interest was performed in 4/7 investigations [38,40–42]. Amelard [31] established the use of temporal correlation of pixel signals with that arterial pulsations in the finger and the technique was used in two subsequent investigations [31,47]. The phase lag between the arterial and venous pulsations identified by Amelard was used to justify a growing spatial clustering algorithm [48], with the venous waveform lagging by approximately 400 ms after the carotid wave [42,43]. Based on frequency domain operations with signal processing, ROIs were refined by the selection of pixels with high power within specific frequency bands [40].

#### Signal processing

*De-trending:* A detrending method [49] was used to remove variations in environmental illumination [31]. One-second means are removed from the pulsatile signal in another approach [42].

*De-noising:* Band-pass and low-pass filters have been used to remove noise, and emphasise the signals within a specific frequency range including ideal bandpass filters and Butterworth filters [20,40,47]. Kalman filters have also been used, based on Newtonian smoothness priors [43,50]. Moving average filters have also been used [42].

*Other processing:* A phased-based Savitzky-Golay gradient correlation algorithm was used for sub-pixel displacement estimation [51]. Eulerian amplification was used on the whole video, with the use of Laplacian pyramids and spatio-temporal bandpass filtering used to emphasise motions within specific frequency ranges [20,52,53].

## 6. Discussion and future direction

All approaches indicate a variety of methods will yield pulsatile signals from the neck. This suggests a camera-based technology is feasible as an assistive device in clinic, or for self-monitoring. Each investigation varied in both hardware and software. As such, a "state-of-the-art" declaration of the technologies so far is futile. This has been a consistent theme in reviews of camera-based technologies thus far [24–26,54,55], though techniques based on neck assessments are more scarce than those based on faces.

### 6.1 Challenges

A multitude of challenges exist for rPPG of the neck, with significant overlap with facial approaches.

#### 6.1.1 ROI selection

Unlike the face, the neck has not had the same attention when it comes to feature detection. As such, segmentation and ROI selection is not as well researched. Compounding this, the neck contains two cardiac pulsatile signals - one from the jugular vein, the other from the carotid artery. The methods used to separate the ROIs included here all rely on the use of external signals from either PPG or ECG to determine heart rate. No method has currently relied solely on the video processing and proved that it is possible to reliably distinguish between the two ROIs.

#### 6.1.2 Motion artifacts

It is generally presumed that the time-varying pixel signal is a direct result of the time-varying light attenuation signal, thereby suggesting a linear relationship. However, this association becomes invalid when the pixel does not measure the exact same area over time, typically due to unrelated subject motion [23,26,56]. Motion artefacts may be due to from various physiological phenomena, such as swallowing, breathing, and head-turning. The magnitude of the effect will likely depend on the region being examined and the processing techniques employed. Swallowing detection may be a necessary problem to solve for this purpose.

#### 6.1.3 Illumination

Lighting conditions may shift over time, leading to the introduction of errors due to shadows or non-homogeneous lighting [23,26,57–59]. Additionally, the relative distance between the camera, participant and light source, significantly impacts the overall signal-to-noise ratio, where SNR decreases with increasing distance [60]. This aspect is particularly relevant when considering the application; for instance, a clinical setting allows for controlled lighting and optimal camera-to-subject distance, thus reducing noise and motion artifacts compared to a system designed for ambient monitoring. However, as the neck’s vessels are deeper than the forehead’s, there may be a requirement for stronger lighting or a closer camera-to-subject distance to capture a discernible pulsatile signal.

#### 6.1.4 Lack of reporting standards

Generally, the development rPPG would benefit from a more systematic approach, including guidelines for experimental design, system design and reporting, as well as publicly available datasets for the benchmarking of processing algorithms. Some guidance regarding this can be drawn from prior reviews concerned with cardiac pulse extraction from other anatomic regions, but the neck consists of two pulsatile signals, the carotid artery and the jugular vein, and so general signal processing approaches may not be adequate for this compared with those derived for the face.

The IDEAL-D and GEP-HI frameworks provide some structure in the development diagnostic systems, including study design and the broader context considerations required for development of novel tools used in medical diagnosis and patient monitoring [61–63]. Using the IDEAL-D framework, the 5/7 investigations thus far are in the Idea phase, Stage 1, with 2/7 investigations in Stage 2a, with iterative developments being used to assess the effects of changes such as patient position or experimental perturbations of pressure.

### 6.2 Design goals

The IDEAL-D framework suggests the development of diagnostic tools are tailored to a specific purpose. As such, hardware and algorithm choices can be chosen to optimise specific outcomes. For example, remote monitoring and telehealth applications will need to develop technology on typical hardware devices such as webcams or smartphone cameras, limiting the utility of complex optical devices (eg. polarizing filters and specialised lighting sources). Software built for this purpose will also likely need to be tolerant to motion and a variety of illumination environments. Conversely, if the system is being developed as a clinical aid, there may not be as much motion or variation in lighting conditions.

### 6.3 Reporting

Regardless of the design goal, standard reporting information should be included. The STARE-HI guidelines provide a good starting point for reporting in journal articles [61,62] and conference papers [64]. We want to develop systems that are equitable and accurate. Equity will include evaluation across all demographics, with accuracy reported. To do so, we need to consider metrics that could impair the accuracy of the system and perform specific evaluation studies on those populations. The reporting of evaluation studies should include detailed demographic and system information for future replication studies to be carried out for populations beyond those previously assessed. Reported demographic information should include any characteristic likely to be seen as either inequitable or could theoretically affect accuracy of the system. For example, sex, age, skin tone, pathologies, body morphology, pharmacology etc. System information should include at least minimum detail for accurate replication, including the make and model of cameras and illumination equipment, participant setup information and ideally the open-source algorithms used, or at least a detailed description of it. Finally, an oft overlooked component to reporting is the reference standard with which the developed system is compared. Reference standards have their own limitations, including racial bias [65,66] and large errors in measurement if the procedure is not performed correctly [66,67]. The gold standard reference for CVP is the right heart catheter. However, the technique for insertion and zeroing can vary, inducing errors [68–70]. For all references used, investigators should, as best as possible, clearly define how the reference device is setup, and quantify uncertainties in the measurement process. In Table 5 we provide our list of recommendations, as well as an fabricated example for illustrative purposes, in a similar fashion to the STARE-HI elaboration publication [62]. Fig. 3 is an illustration of the example provided in Table 5.

**Table 5.** Recommendations for minimum reporting guidelines. The example provided is fabricated, for the illustration of how this may be reported. The use of [citation] and [table] indicate that a citations and tables should be included, providing a link to the model information if possible.

Section	Parameter	Fabricated Example	Elaboration
**Study**	Objectives	"The objective of this study is to investigate the role of skin tone in the SNR of the cardiac pulsatile signal in the neck in RGB cameras for participants in the waiting room of the Emergency Department."	The objective of the study should be clearly stated, including the **aim**, **metric**, **system**, and if relevant the **population** involved.
	Setting	"This study was carried out in the Emergency Department of a tertiary hospital in Buenos Aires, Argentina. "	For real-world studies, investigations of how the system may be integrated in a clinical workflow, it is important to note the **setting** and **geographic location** of the study. This provides context regarding how systems may be implemented and their effectiveness in other clinical contexts. For laboratory based studies, it may be relevant to note lighting variations due to windows.
	Demographics	"The study included 50 participants (30 male), aged between 16 - 55 years ( μ : 39 σ : 8). Skin tone was assessed via the monk skin tone scale, with skin tones ranging from 3 - 8 ( μ : 4.5 σ :1.9). 4 participants had diagnosed arrhythmias. A detailed breakdown of the demographic data can be found in [table]."	Demographic data should include at a minimum, **age**, **sex**, **skin tone**, and any **relevant pathologies and pharmacology**. A detailed table should be included with a breakdown of the demographic information.
	Setup	"Participants sat vertically, with heads facing forwards. The neck was cleaned with alcohol wipes prior to recording to remove any residual covering such as makeup. The camera was positioned at a distance of 30 ± 10 cm from the neck, at an angle of approximately 45°from the face. The pulse oximeter was affixed to the index finger of the participant of their dominant hand. With the flash on, a 10 second video was recorded, with synchronous acquisition of the pulse rate via the pulse oximeter.	The setup of the system, including how the **reference standard** is used, should be documented for future replication studies. This should include a **description of the lighting**, **camera-patient position**, and **patient position** (eg. supine, sitting, recumbent).
	Reference Standard	"The ground-truth heart rate was measured via a bluetooth pulse oximeter (*Heart Sure Bluetooth Pulse Oximeter* [citation]) to collect synchronous pulse rate information with the video".	Reference standards also have uncertainties associated with their measurements. Ideally, these are quantified in the publication, however, **make** and **model** of the equipment are sufficient.
**Hardware**	Camera	"An *iPhone 14 Pro* [citation] was used for the video recordings. RGB recordings were made, at 60fps with auto-exposure and 4k resolution."	The **make** and **model** of the camera should be mentioned. Further camera parameters such as **colour channels** used, **exposure settings** and the **frame rate**.
	Illumination	"The iPhone flash was used with a custom diffuser on the flash made using white printer paper. At a distance of 30 cm, the illuminance is 1200 ± 100 lux, with the spectra shown in Figure 3. The illuminance and spectra was verified using an *Oppel Light Master 3* [citation]."	Lighting should be mentioned explicitly. Ideally, the lighting should be quantified, with **illuminance** and/or **spectra** being given. If this is not possible, the **make** and **model** of the corresponding lamps should be recorded. This is required for the use of non-standard lighting (such as the use of coloured LEDs). For clinical settings, attempts at quantification or general lighting conditions should be noted (i.e. fluorescent globes).
	Other Optical Devices	"A circular polarising lens was fitted to the phone camera (*Moment M-Series 37mm Cine CPL [citation]*)."	**All other optical devices** such as diffusers, wavelength and filters should be recorded.
**Software**	Pipeline and Algorithms	"For each video, a gaussian blur of 20x20 pixels was applied across each of the colour channels. Iterating through each pixel, a fourier transform was applied to each colour channel, and the SNR was calculated for each pixel, using the reference standard as the ground truth [71]. The SNR for each pixel was taken to be the mean across the 3 colour channels. The top 10% of pixels was recorded, and the mean, median and standard deviation of these pixels was used as the index for total SNR in the video. The code for the processing, as well as the de-identified data can be found at https://github.com/example/example_respository."	We should include **all the steps in processing**, including the **calculation of any metrics** used in the analysis. Ideally, the **processing algorithms** will be made publicly available. If not, they should be described in sufficient detail such that replication is possible. For **AI models, the trained model** should be made available for future evaluation.

### 6.4 Public datasets

A commonly referenced shortcoming in the previous reviews is the limited publication of public datasets, particularly for people with pathologies [22–26,55]. The primary region of interest in most literature is the face. An unfortunate shortcoming with facial videos is that they by necessity contain identifying features. The neck provides a region of interest with strong pulsatile signals, information regarding both the right and left side of the heart by means of the jugular veins and the carotid arteries, without the limitation of easily identifiable features. This provides strong motivation for the future of large-scale datasets containing the neck as a region of interest, instead of the face.

### 6.5 Benchmarking and design choices

Benchmarking consists of the establishment of a baseline with which future investigations can be compared. A benchmark system can be established in two forms: hardware and processing. Hardware benchmarks establish minimum system requirements for the cameras and lighting used and can be compared with via benchmark algorithms. Benchmark algorithms establish themselves on multiple datasets to ensure accuracy in a variety of applications. The number of investigations into the neck as a whole limit the ability of the authors to establish benchmarks for systems or algorithms, however, guidance can be provided from parallel research into other camera-based investigations into vital sign estimation. The results are summarised in Table 6.

**Table 6.** Hardware guidance based on general remote photoplethysmography analyses.^*a*^

Hardware	Parameter	Recommendation
**Camera**	Frame Rate	Minimum frame rate used should be 20 FPS. Increases in frame rate will likely increase accuracy of peak detection
	Colour Channels and Colour Filter Array	Colour channels should be selected depending on the purpose. Shorter wavelength increase signals reflected from the surface of the skin, and longer wavelengths will penetrate deeper.
	Bit Depth	Increases in bit depth will likely increase SNR, particularly for PPG based approaches. It may be useful to increase accuracy of peak detection.
	Resolution	Resolution requirements are dependent on the camera-subject distance. For close applications, resolution of 720p as a minimum would be a suitable option.
	Compression	Compression algorithms significantly reduce accuracy in heart rate detection. As such, compression should be minimised prior to processing.
**Illumination**	Brightness	All applications thus far perform better with increased illumination for the corresponding sensor.
**Other Optical Devices**	Polarizing Filters	Polarizing filters can be used to explicitly select for specular or diffuse reflection. For insight-based research, this is important. It’s unclear as to how much the SNR will change with the addition of these filters. In the development of wide-scale technology, it may introduce undesirable complexity.

^*a*^ FPS: Frames per second PPG: Photoplethysmography SNR: Signal to Noise Ratio

#### 6.5.1 Hardware

##### Camera

*Camera Resolution and Camera-Subject Distance:* Visual realisation of pulsatile signals in the neck are inherently difficult for individuals, as evidenced by the frequent inability of clinicians with no vision defects, to realise the pulsatile signal at short ranges. It is therefore likely that this would benefit from superhuman fidelity via the use of cameras. Signal extraction from videos relies on the number of pixels expressing that signal. As blood pulse signals are relatively noisy within a single pixel, increasing the number of pixels for signal extraction can significantly reduce the amount of noise. Investigations by Song *et al.* into the effect of camera resolution showed that for any distance greater than 0.5 m, increasing the camera resolution reduces the error in heart rate measurement across a variety of signal extraction techniques [72]. For distances less than 1.5 m, resolution of at least 720p can result in heart rate accuracy within 4 BPM (beats per minute).

*Frame rate:* Cardiac pulsatility can be assumed to occur for most people between 30 – 240 BPM (0.5 - 4 Hz). The Whittaker-Nyquist-Shannon sampling theorem [44–46], suggests a minimum sampling frequency of twice the maximum frequency is required to reconstruct the original signal and avoid aliasing, so cameras with > 8 FPS should be able to accurately reconstruct the cardiac cycle. Indeed, Blackford and Estepp found no degradation in signal processing when down-sampling from 120 FPS to 30 FPS [73]. This finding was challenged by Speth *et al.* using a convolutional neural network, where the mean absolute error increased from <4.18 BPM to >6 BPM for frame rates less than 11.3 FPS [74].

*Colour Channel and Colour Filter Array:* Monte-Carlo modelling of light for reflectance PPG devices such as smart watches show that longer wavelengths have increased depth penetration as compared with shorter wavelengths [75]. As such, the signals from IR cameras like those used by Amelard *et al.* [31,43] are a result of light interactions with greater tissue volumes, and at greater tissue depth than blue or green light. The arrangement of the bandpass filters over the charge-coupled device (CCD) used to get RGB images is termed the colour filter array. Typically a Bayer array is used, where the ratio of G:R:B filters is 5:2:2, mimicking the human eye and its increased sensitivity to green light [76]. From use of standard RGB sensors, it has been shown that green light has the highest signal to noise ratio [58]. McDuff *et al.* investigated the use of 5 colour channel cameras and their impact on pulse measurement and breathing rate [77]. The 5 channels were red, green, blue, cyan, and orange (RGBCO). They found that by using CGO increased the measurement accuracy of heart rate variability and that no benefit was obtained from the red or blue channels, and that the addition of the blue channel degraded performance. The addition of an orange colour channel was noted to be particularly informative. This could be due to the combination of increased tissue depth penetration of green and orange light compared with blue, and the peak absorption of the orange spectra at 580 nm, corresponding closely with one of the peaks of the absorption spectra of the two dominant chromatophores in blood, oxyhemoglobin and deoxyhemoglobin [78]. To the best of our knowledge there have been no investigations into the effects of colour filter array patterns. However, this information flows downstream in terms of variation in illumination and spread of measured values, and so assessments of this may not be necessary.

*Bit Depth:* Bit depth refers to the detail in the recorded intensity. The parallel could be made to significant figures, or resolution of a measurement device. Cameras typically capture values for each channel in 8-bit depth, giving values between 0-255. Bit depths of 10, 12 and 14 have been used in previous investigations [37,79–82]. Due to the variation in processing and site selection, the effect of increasing bit depth has not yet been defined. However, as identified by McDuff *et al.* [77], under non-ideal conditions, most cameras successfully can ascertain heart rate, however, peak detection is limited, and so parameters such as heart rate variability are more difficult to measure accurately. By increasing bit depth, this may improve the temporal accuracy of peak detection, and may warrant investigation, especially with the association between heart-rate variability and blood pressure [83].

*Image Compression:* Cameras typically compress the captured videos for faster file transfer and reduced storage space. Several investigations have been conducted into the various video compression techniques and video encodings [84–86]. All investigations showed that with increasing video compression, accuracy of heart rate extraction decreased. A variety of compression algorithms exist, and the magnitude of the effect will likely be different for each of them. For instance, as the PPG signal is primarily due to chromaticity variations, chroma-subsampling as performed in the YUV colourspace is likely to reduce the SNR of the measured signal for each pixel. Similarly, micro-motion of the skin contributes to the signal via specular reflection, and algorithms that may stabilise or remove this motion will further deteriorate the signal [85].

##### Illumination

The measured signal is highly dependent on the illumination of the surface. As such, illumination is likely to play a significant role in the accuracy of the measured signal. Illumination in studies thus far is either defined as indoor or artificial light such as those in a lab, hospital, or office, natural/ambient as provided by the sun, or specifically designed to elucidate specific features that may be useful for specific wavelengths, such as blue light used by Moço *et al.* [41] when attempting to enhance the surface motion [26].

##### Other optical devices

*Filters:* If an artificial lighting source is used to exaggerate specific signal properties, filters can act to further highlight those properties. By using bandpass filters that correspond with the spectrum of the light, we can then probe specific depths within the tissue. For instance, if we use an infrared filter, the reflected signal will be a result of interactions at deeper tissue depths compared with that of a blue filter. This is particularly useful when attempting to visualise noisy signals at depth, such as those of venous pulsations, and was employed by Amelard *et al.* [31,43]. Monochromatic cameras often allow higher frame rates, at the expense of loss of colour information, and so a monochromatic camera may have higher frame rate and resolution than an RGB camera at the same price point. By using filters with monochromatic cameras, one can selectively remove wavelengths which may be less useful (eg. red signals may have higher noise due to motion artifacts [87]), while retaining the benefit of the increased frame rates and reduced camera cost.

*Polarizers:* Polarizing filters on both the light source and sensor reduce specular reflection component in the signal. When light is oriented orthogonal to a surface with a linear polarizing filter it results in negligible specular reflection. By combining this with a circular polarizing filter on the image sensor, one can ensure that the signal measured is due to blood volume changes (i.e., PPG) effects below the surface. Thus, using polarizing filters, we can increase specific signals either due to PPG effects, or surface effects [26,88].

#### 6.5.2 Software

##### Signal processing approaches

Traditional signal processing approaches have been investigated significantly in parallel literature and other reviews [26,55]. This includes ROI selection by automated segmentation, ROI tracking algorithms for motion tolerance, spatial filtering to reduce quantization noise, detrending and bandpass filters to emphasise specific desired frequencies, wavelet transforms and more. The algorithms proposed have been assessed on a range of publicly available datasets, which is unable to be performed in this instance, and so a "best approach" recommendation is not possible. A publicly available dataset consisting of neck based videos and corresponding heart rate wave forms would be a beneficial to this end.

##### Artificial intelligence and machine learning

Artificial intelligence (AI), machine learning and deep learning algorithms are emerging as being more accurate than traditional signal processing approaches for heart rate detection among faces [28]. Furthermore, AI can be used to automatically segment and define the ROI for traditional approaches [89], or traditional approaches can supply a signal to an AI algorithm for future processing [41]. This promising application is yet to be investigated on any neck-based assessments, however, this has been used widely in contact-based finger, and wrist based photoplethysmography systems for the purposes of measuring arterial blood pressure [88–90]. A recent comparison of several algorithms was recently conducted, and found that Support Vector Regression (SVRs) may marginally outperform other architectures [84].

### 6.6 Limitations of review

Due to a limitation in resources, this systematic review was limited to a single-reviewer model for the screening and review of articles, which, although methodical, introduces the potential for selection bias. The scope of this review is further constrained by the focus on English-language publications, potentially omitting significant findings reported in other languages. The review was limited to investigations of the jugular vein, and so by doing neglects potential insights drawn from investigations concentrating on the carotid arteries. Due to the shared challenge of ROI selection, future reviews would do well to include both. This presents an analytical challenge for distinguishing between these two sources of signals. To address this, future studies could benefit from a dedicated review of literature focusing on the discrete analysis of signals from both vascular structures.

It is worth noting that the fields of artificial intelligence, deep learning, and neural networks are rapidly advancing. As such, techniques related to ROI selection and segmentation that employ these computational models are likely in nascent stages of development. Although the current body of literature in this domain is limited, we anticipate that future work will explore these avenues, providing more sophisticated methods for signal analysis.

## 7. Conclusion

This review details the approaches used to measure the jugular venous pulsations in the neck. The utility of the jugular veins is well established and continues to provide clinical utility. Several techniques have been used to accurately show pulsatile signals and large pressure changes in the neck. We have summarised the design of previous studies by their demographics (Table 1) and, experimental setup (Table 2), with results of these investigations shown (Table 3). The technology is now at a stage where it would benefit from some of the resources that exist for general camera-based assessment of vital signs, including published datasets [91–94] and a repository of benchmarking algorithms [55,92] to facilitate future investigations.

This review showed that a variety of approaches are all feasible with variations in both hardware (Table 4) and processing techniques, but that a comparison between the approaches is not yet possible. To allow comparisons in the future, the field requires standardisation in reporting, and publicly available datasets. We provide guidance regarding minimum reporting information for future systems with an illustrative example (Table 5). Hardware suggestions are provided to assist new investigators in the field (Table 6). Finally, we envisage the role of AI and machine learning algorithms in the development of automatic ROI segmentation, diagnosis of arrhythmias and the absolute estimation of pressure from imaging systems.

## Data Availability

No data were generated or analyzed in the presented research.
